# Obestatin treatment links mitochondrial homeostasis and skeletal muscle repair in Duchenne muscle dystrophy

**DOI:** 10.1186/s43556-025-00370-8

**Published:** 2025-11-25

**Authors:** Andrea C. Lodeiro, Silvia Costas-Abalde, Tania Cid-Díaz, Lucía Debasa-Corral, Saúl Leal-López, Kamel Mamchaoui, Vincent Mouly, Xesús Casabiell, Rosalía Gallego, José Luis Relova, Yolanda Pazos, Icía Santos-Zas, Jesus P. Camiña

**Affiliations:** 1 Grupo de Miología, Instituto de Investigación Sanitaria de Santiago (IDIS), Complejo Hospitalario Universitario de Santiago (CHUS), Servicio Gallego de Salud (SERGAS), Trav. Choupana S/N, Santiago de Compostela, Spain; 2 Grupo de Investigación Traslacional en Enfermedades del Aparato Digestivo (GITEAD), IDIS, CHUS, SERGAS, Trav. Choupana s/n, Santiago de Compostela, Spain; 3 Institut de Myologie, Centre de Recherche en Myologie, Sorbonne Université, Inserm, 75013 Paris, France; 4 Departamento de Fisiología, Universidade de Santiago de Compostela (USC), Santiago de Compostela, Spain; 5 Departamento de Ciencias Morfológicas, USC, Santiago de Compostela, Spain

**Keywords:** Duchenne muscular dystrophy, Obestatin, Mitochondria, Utrophin, TFEB, NFATc1

## Abstract

Duchenne muscular dystrophy (DMD) is a genetic, progressive neuromuscular disease caused by mutations in the dystrophin protein which compromise the integrity of the sarcolemma. Current care of DMD involves both supportive and targeted disease modifying medications. Obestatin, a peptide derived from preproghrelin, is a potential candidate to enhance existing treatments for DMD. This study was conducted to analyse the molecular mechanism by which obestatin acts on myofiber metabolism and muscle restructuring in DMD. Through human and animal models of DMD, we identify the calcium-activated protein phosphatase 3 (PPP3) as key node in obestatin signalling for restoration of muscle homeostasis and activation of membrane repair. In particular, we describe how obestatin signalling recovers muscle function by coordinated activation of the transcription factor EB (TFEB) and the nuclear factor of activated T cell (NFATc1) in which PPP3 is a core component. TFEB dephosphorylation triggers its nuclear translocation and the activation of macroautophagic/autophagic and mitochondrial biogenesis. NFATc1 promotes the slow myofiber phenotype fibre marker utrophin. Overall, obestatin treatment ameliorates distinctive dystrophic features of DMD, including muscle contractile damage, elevated serum creatine kinase levels, and reduced muscle force. Hence, obestatin represents a promising therapeutic approach for treating DMD, not only as monotherapy but also as part of combinatorial treatment strategies aimed at overcoming the barriers that limit the efficacy of gene or cell therapy.

## Introduction

Duchenne muscular dystrophy (DMD) is caused by pathogenic variants in the X-linked DMD gene, resulting in the absence of functional dystrophin and continuous muscle damage, starting at birth [1]. Impaired motor function is evident by the age of three, and during adolescence, it usually progresses to loss of ambulation with standard corticosteroid treatment [1, 2]. Muscles lacking dystrophin are vulnerable to injury, leading to a progressive loss of muscle tissue and function, in addition to cardiomyopathy. A multidisciplinary medical, surgical and rehabilitative approach addressing the symptoms of DMD is now able to change the natural course of the disease, which results in a better quality of life and, consequently, increased longevity. Nonetheless, DMD remains an incurable condition despite considerable therapeutic advancements over the past 30 years. A number of therapies aimed at either restoring dystrophin protein or addressing secondary DMD-related issues, have obtained regulatory approval and several others are in clinical development [2–6]. Thus, to date, there is an unmet demand for treatments that can more accurately stabilise or decrease the course of the disease and that may be appropriate to all forms of DMD [2].

In addition to the therapeutic strategies targeting dystrophin, significant efforts have also been made to target the consequences of dystrophin deficiency to preserve muscle tissue for an extended duration [7, 8]. Compared to gene-directed and cell-based therapies, pharmacological strategies offer a key advantage: broad applicability and safety, irrespective of the type of mutation. In addition, the efficacy of both therapeutic approaches relies on the quality of the muscle to reduce disease progression as far as possible [9]. In this sense, we previously demonstrated the potential of obestatin, a 23-amino acid peptide derived from the preproghrelin polypeptide, as a therapeutic agent targeting skeletal muscle myopathies involving muscle degeneration/regeneration in addition to muscle injury [10–12]. Remarkably, the obestatin system controls myogenesis through an autocrine function [10–12]. This action is determined by the G-protein-coupled receptor GPR39 that transactivates the epidermal growth factor receptor (EGFR) through the β-arrestin signal complex. The cross-talk between GPR39 and EGFR delineates the cell cycle exit and myogenic differentiation via the Akt, c-Jun, and p38 axis [10–12]. The balance between G-protein-dependent and β-arrestin-dependent signalling pathways determines the progression of the myogenic program. In addition to its function in myogenesis during muscle regeneration, obestatin plays a role in determining muscle fibre type, promoting an oxidative muscle phenotype [13]. Obestatin controls the establishment of oxidative muscle fibres through both class II histone deacetylases/myocyte enhancer factor-2 and peroxisome proliferator-activated receptor-gamma coactivator 1α (PGC1α). As an anabolic system, obestatin counteracts proteostasis deregulations, such as those associated with glucocorticoid-induced atrophy, and restores basal homeostasis in an efficient manner [14, 15]. Both transcriptional and post-translational FoxO activities are regulated by obestatin signalling. At transcriptional level, the upregulation of the E3 ubiquitin-protein ligase NEDD4, which determines the ubiquitin-dependent degradation of Krüppel-like factor 15, regulates the expression of FoxO transcription factors. At post-translational level, the modification of FoxO transcription factors, including the phosphorylation of FoxO4, plays a pivotal role in the response to obestatin signalling. Simultaneously, obestatin positively modulates myofibre size via mTOR signalling, thereby restoring muscle phenotype in glucocorticoid-treated mice. Furthermore, obestatin restores muscle integrity and partially rescues muscle tissue necrosis in a DMD murine model [16]. In addition to slowing muscle damage, obestatin has significant therapeutic potential as a component of combined therapeutic strategies to achieve greater efficacy than a single treatment alone. In cell transplantation therapy, for example, obestatin improves both the efficacy of the graft and the uniform distribution of myoblasts within the host muscle by promoting migration [13]. These findings support the applicability of obestatin to treat both muscle injuries and skeletal muscle myopathies involving muscle degeneration and/or regeneration.

There is currently great interest in the development of combinatorial therapies for DMD treatment. Such strategies should be designed to treat the secondary consequences of muscular dystrophy that worsen the efficacy of individual therapies, such as muscle degeneration, inflammation, or fibrosis. It is therefore essential to integrate the positive results of different therapies that focus on diverse aspects. In this study, we examined the impact of obestatin on myofibre metabolism, muscle restructuring, and muscle function in human and animal models of DMD. We hypothesize that proper reactivation of mitochondrial homeostasis, lysosomal function and sarcolemma repair in dystrophic muscles would replenish the muscle fibres with functional organelles and proper structure, alleviating the symptoms of DMD disease.

## Results

### Evolution of mitochondrial homeostasis in *mdx* mice with age

**Fig. 1:**
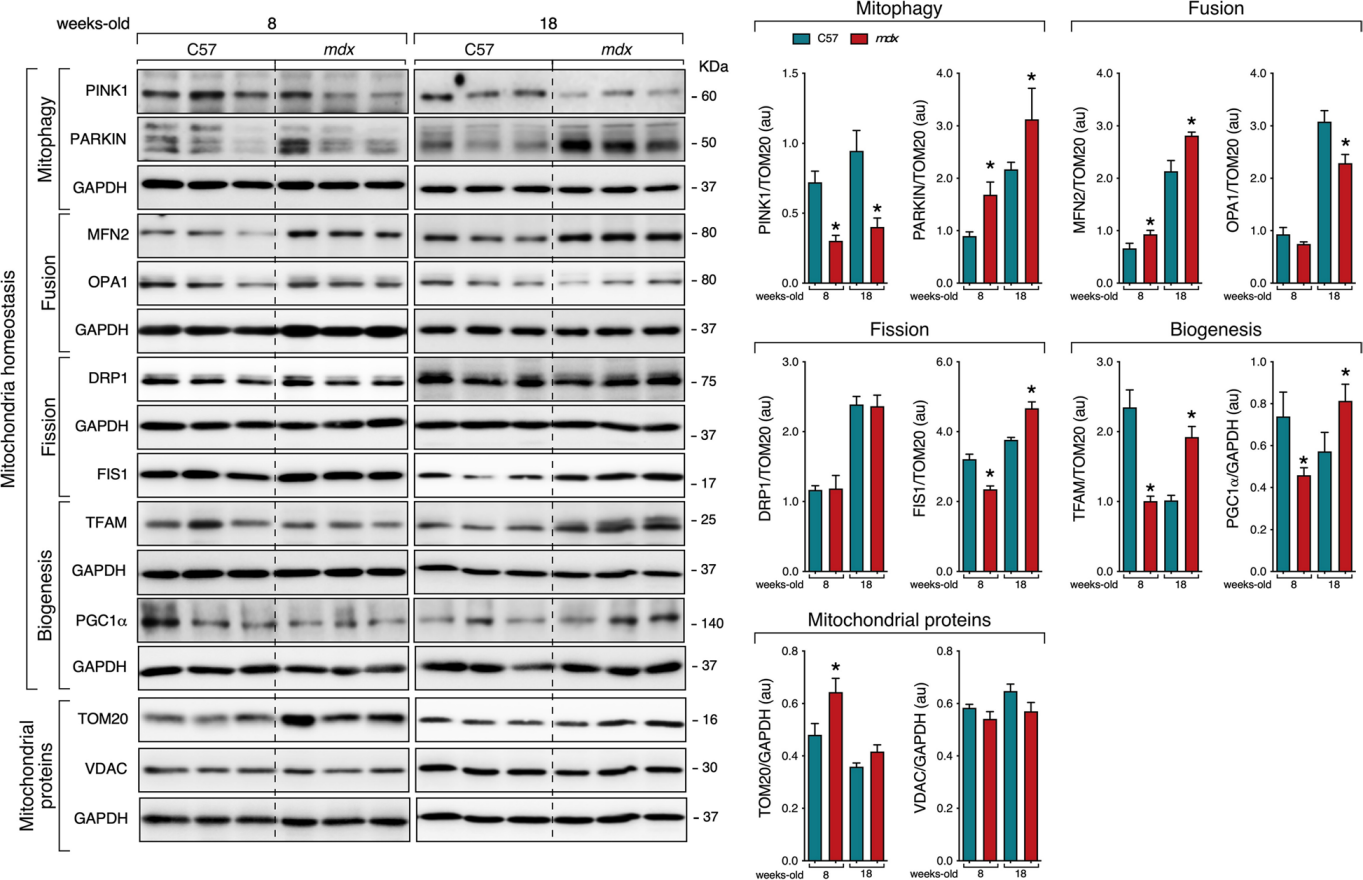
Altered basal mitochondrial homeostasis in DMD skeletal muscle. The figure refers to the TA muscles of C57BL/10ScSn and *mdx* mice of different ages (8-, and 18-week-old C57BL/10ScSn and *mdx* mice; *n* = 3 per age group). *Right panel*, Immunoblot analysis of PINK1, PARKIN, MFN2, OPA1, DRP1, FIS1, TFAM, PGC1α, TOM20, and VDAC in the TA muscles. Protein level was normalized to TOM20 or GAPDH as indicated. Data were expressed as the mean ± SEM (*P < 0.05)

The mitochondrial dynamic in the context of DMD was first examined in tibialis anterior muscle (TA) of C57BL/10ScSn-Dmdmdx/J (*mdx*) at 8-week-old mice, which represent the active regeneration phase of the disease (prior to the severe muscle damage, apoptosis and necrosis), and compared them to age-matched C57BL/10ScSn (healthy control) mice. When normalised to the mitochondrial content by using translocase of outer mitochondrial membrane 20 (TOM20) as mitochondrial marker, the levels of PTEN-induced kinase 1 (PINK1), mitochondrial fission protein 1 (FIS1), mitochondrial transcription factor A (TFAM) and PGC1α protein showed a decline in *mdx* mice relative to age-matched controls. Both E3 ubiquitin-protein ligase parkin (PARKIN) and mitofusin 2 (MFN2) were significantly increased, but no changes of dynamin-related protein 1 (DRP1) and voltage-dependent anion-selective channel (VDAC) were observed (Fig. 1). These data imply the alteration of mitochondrial life cycle at early phase of regeneration of the disease. Our analysis was extended to 18-week-old mdx mice, which represents the late stage of degeneration phase, and compared to age-matched control mice. Similarly, we observed a decrease in PINK1 while the protein level of PARKIN and MFN2 increased in *mdx* vs. age-matched controls (Fig. 1). Despite observing no differences in DRP1, TOM20 and VDAC protein levels, we found increased levels of FIS1, TFAM and PGC1α in 18-week-old *mdx* relative to age-matched controls (Fig. 1). Thus, *mdx* muscle exhibits impaired mitochondrial homeostasis simultaneously with muscle fibber damage that contributes to abnormal cellular fate in this pathology.

### Modulation of mitochondrial dynamic by obestatin in *mdx* mice

**Fig. 2:**
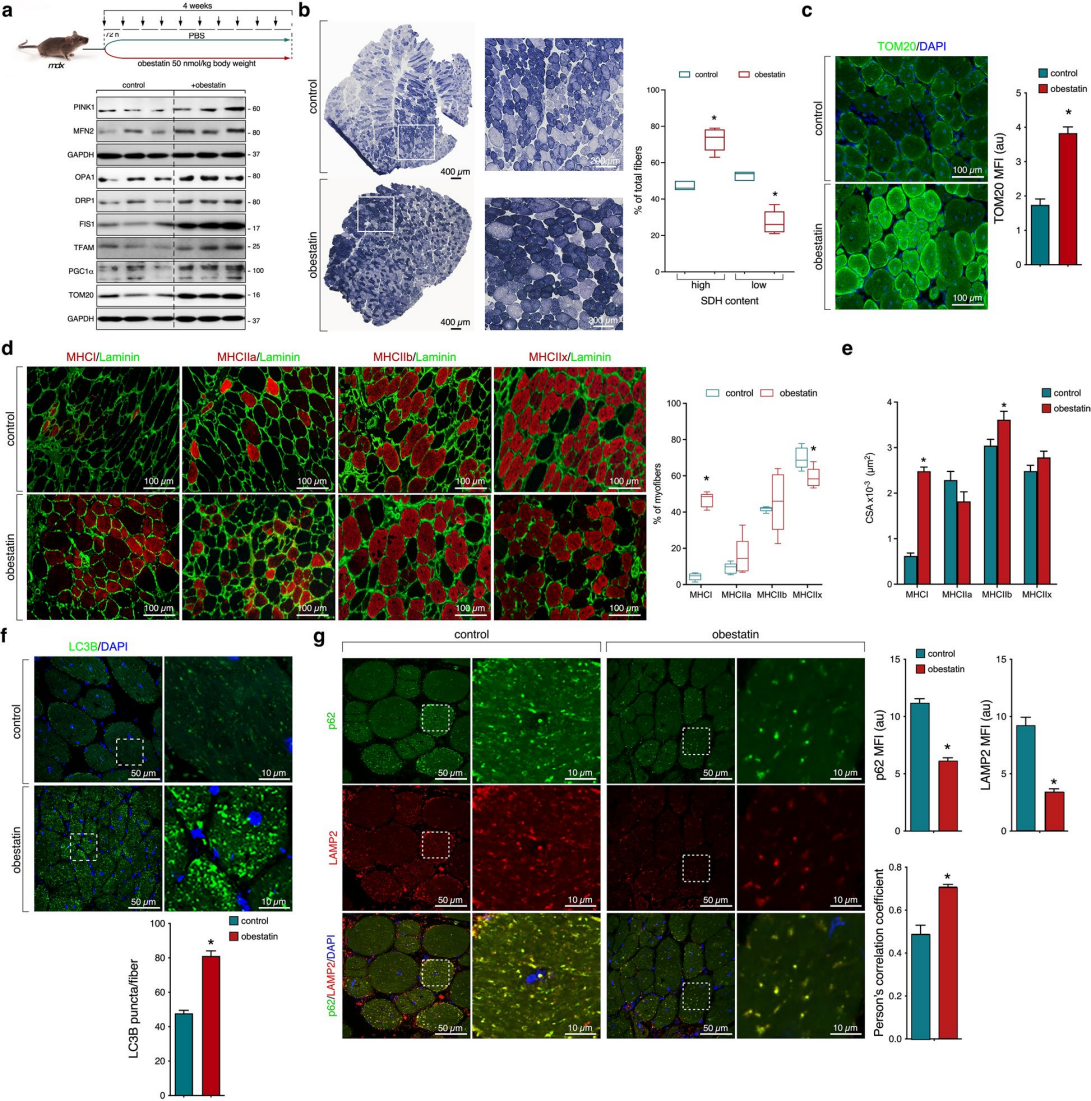
Activation of mitochondrial and autophagic dynamic by obestatin treatment in *mdx* mice. **a** *Upper pan*el, schematic diagram of intramuscular administration of vehicle (control) or obestatin (500 nmol/kg each 72 h) in the TA from *m*dx mice. *Lower panel*, protein levels of PINK1, MFN2, OPA1, DRP1, FIS1, TFAM, PGC1a, and TOM20 in vehicle- and obestatin-treated TA muscles at 30 days (*n* = 5 per group). **b** Representative SDH staining from TAs at day 30. *Right panel*, quantitation of oxidative (high SDH content) and glycolytic (low SDH content) muscle fibbers. Line is set at median. *P < 0.05. **c** Representative images of vehicle- and obestatin-treated TAs showing TOM20 expression. The changes in mean fluorescence intensity (MFI) of TOM20 are shown (*n* = 5 per group). **d** Representative images of MHCI, MHCIIa, MHCIIb, or MHCIIx expression in TAs from *mdx* mice. *Right panel*, quantitation of fibre types in TAs. Line is set at median. *P < 0.05. **e** Quantification of CSA in TAs from *m*dx mice. **f** Representative images of vehicle- and obestatin-treated TAs showing LC3B expression. *Bottom panel*, quantification of LC3B puncta per TA fibre. **g** Representative images of vehicle- and obestatin-treated TAs showing p62 and LAMP2 expression. The changes in MFI of p62 and LAMP2 are shown. Pearson’s coefficient (r) indicates the correlation of intensity values of p62 (green) and LAMP2 (red) pixels in dual-channel images. In B-G, data were expressed as mean ± SEM. *P < 0.05 vs control values (*n* = 5 per group)

Given the impaired mitochondrial dynamics, we hypothesized that obestatin, a peptide capable of stimulating AMPK under dystrophic conditions, could restore these mitochondrial defects in DMD. We administrated obestatin to *mdx* mice via intramuscular injection into the TA muscles every 72 h (h) during 30 days [500 nmol/kg body weight (*n* = 5); Fig. 2a], starting at 8 weeks of age. The results from this group were compared with those from vehicle-treated *mdx* mice [phosphate buffered saline (PBS; *n *= 5); control group] under the same conditions. Obestatin treatment was associated with increased PINK1, MFN2, OPA1, DRP1, FIS1, TFAM, PGC1α and TOM20 protein levels which support a role in the control of mitochondrial homeostasis under dystrophic condition (Fig. 2a). To evaluate mitochondrial activity, we measured the intensity of succinate dehydrogenase (SDH) staining. When compared to control *mdx* mice, obestatin-treated TA showed a higher density of oxidative fibres characterized by high SDH concentration (Fig. 2b). In fact, obestatin-treated TA exhibited an increase in the total amount of mitochondria (TOM20) (Fig. 2c). As mitochondrial dynamics are essential intracellular platforms to differentiate slow and fast fibres [17], we studied if the proportion of slow fibres was modified by means analysis of specific myosin heavy chain (MHC) isoforms in TA muscles. Specific immunostaining against MHC type I (MHCI) revealed that obestatin-treated *mdx* mice showed more MHCI than control mice (Fig. 2d). These TAs had a decrease in type IIx fibres related to control, whereas no significant changes were identified in type IIa and IIb fibres (Fig. 2d). When compared to normal *mdx* mice, the cross-sectional area (CSA) of type I fibres was significantly higher in obestatin-treated TAs (Fig. 2d). Type IIa and IIx fibres showed no significant differences, but type IIb fibres exhibited a significantly larger CSA in obestatin-treated mice compared to control mice (Fig. 2e). Mitochondrial function is known to be dependent on the proper functioning of autophagy [18]. Notably, obestatin-treated TA muscles exhibited marked increase of microtubule-associated proteins 1A/1B light chain 3B (LC3) immunofluorescent staining after obestatin treatment (Fig. 2f), and a decline in the fluorescent signal of ubiquitin-binding protein p62 (p62) indicating autophagic degradation activity compared with control group (Fig. 2g). This degradation activity was confirmed by a down-regulation of lysosome-associated membrane protein 2 (LAMP2) levels in obestatin-treated *mdx* mice compared to control *mdx* mice (Fig. 2g). Noteworthy, the dissipation of p62-formed aggregates and LAMP2-positive inclusions after obestatin treatment indicated features of efficient autophagic flux (Fig. 2g).

### Validation of obestatin action on mitochondrial dynamic and autophagy in human immortalized DMD myoblasts

**Fig. 3:**
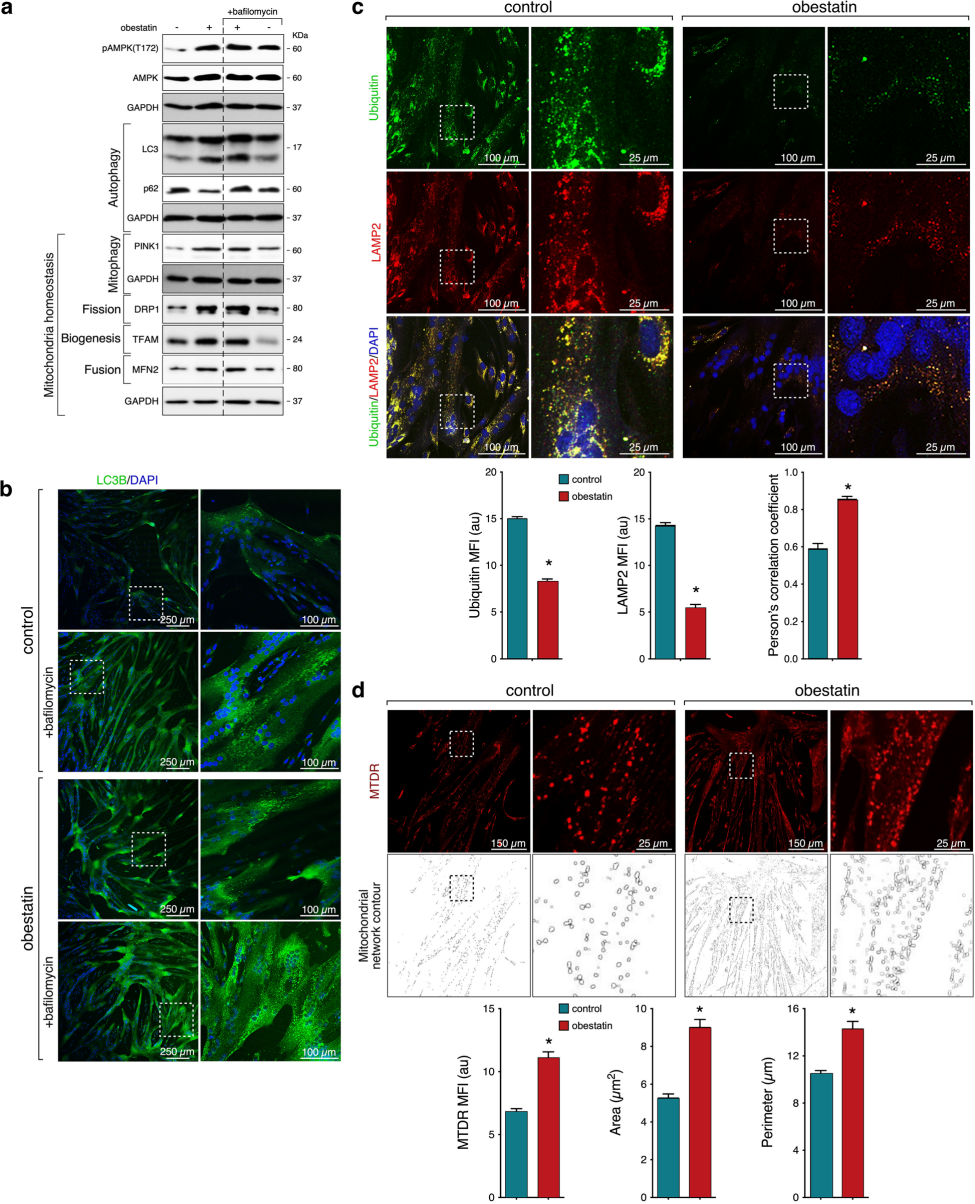
Obestatin rescues autophagy flux and mitochondrial homeostasis in human DMD myotubes. **a** Human DMD myotubes were treated with obestatin (10 nM) in the presence or absence of bafilomycin A1 (100 nM, 4 h pretreatment). pAMPK(T172), AMPK, LC3, p62, PINK1, DRP1, TTFAM, and MFN2 were analysed by western blot. Immunoblots are representative of the mean value. **b** Representative images from LC3B immunostaining of DMD myotubes treated with obestatin (10 nM) or vehicle (control) in the presence or absence of bafilomycin pretreatment. **c** Representative images from ubiquitin and LAMP2 immunostaining of human DMD myotubes treated with obestatin (10 nM) or vehicle (control) for 24 h. The changes in MFI of ubiquitin and LAMP2 are shown. Pearson’s coefficient (*r*) indicates the correlation of intensity values of green and red pixels in dual-channel images. **d** MitoTracker Deep Red.® (MTDR) staining and corresponding mitochondrial network contour of human DMD myotubes exposed to vehicle (PBS) or obestatin (10 nM) to assess mitochondrial analysis. *Bottom panel*, the changes in MFI of MTDR as well as the scoring of mitochondrial morphology, area (the space occupied by a mitochondrion) and perimeter (the outer boundary of mitochondrion), of the mitochondrial network are shown. **c-d**, data were expressed as mean ± SEM (*n* = 5 per group; *P < 0.05)

We next validated the activation of autophagy by obestatin in an in vitro model of human DMD skeletal muscle cells. In these cells, an increase in the activation of AMPK [pAMPK(T172)] and the conversion rate of the LC3I to LC3II was observed concomitant with reduced accumulation of p62 (Fig. 3a). Bafilomycin, which prevents fusion between autophagosomes and lysosomes, increased the LC3II and p62 accumulation (Fig. 3a), confirming the autophagic flux activation. Obestatin increased the levels of PINK1, DRP1, TFAM and MFN2, underlining its effect on mitochondrial homeostasis in this context (Fig. 3a). Accumulation of LC3 immunostaining was detected in obestatin-treated DMD myotubes, which was sensitive to bafilomycin treatment (Fig. 3b), supporting the activation of autophagy in obestatin-treated myotubes. These findings were reinforced by experiments in which DMD myotubes were immunostained for ubiquitin and LAMP2 (Fig. 3c). The decrease in ubiquitin signalling and its colocalization with LAMP2 supported that the autophagic defect was partially, at least in part, overcome by obestatin signalling through restoration of autolysosomal clearance (Fig. 3c). Remarkably, the up-regulation of mitochondrial homeostasis-related proteins was accompanied by an enhancement in the mitochondrial network (Fig. 3d). This fact supported the ability of obestatin to reverse the mitochondrial phenotype associated to DMD. Indeed, the presence of myotubes with high values of MitoTracker Deep Red® (MTDR) is an indication of correct mitochondrial function, typically associated with gain of mitochondrial membrane potential, inhibition of apoptosis, or cellular stress [19]. Thus, these findings suggest that obestatin signalling improves the mitochondrial homeostasis and partially overcomes the autophagic defect under DMD conditions.

### Obestatin action converges on restructuring of muscle fibre and improvement of muscle function in *mdx* mice

**Fig. 4:**
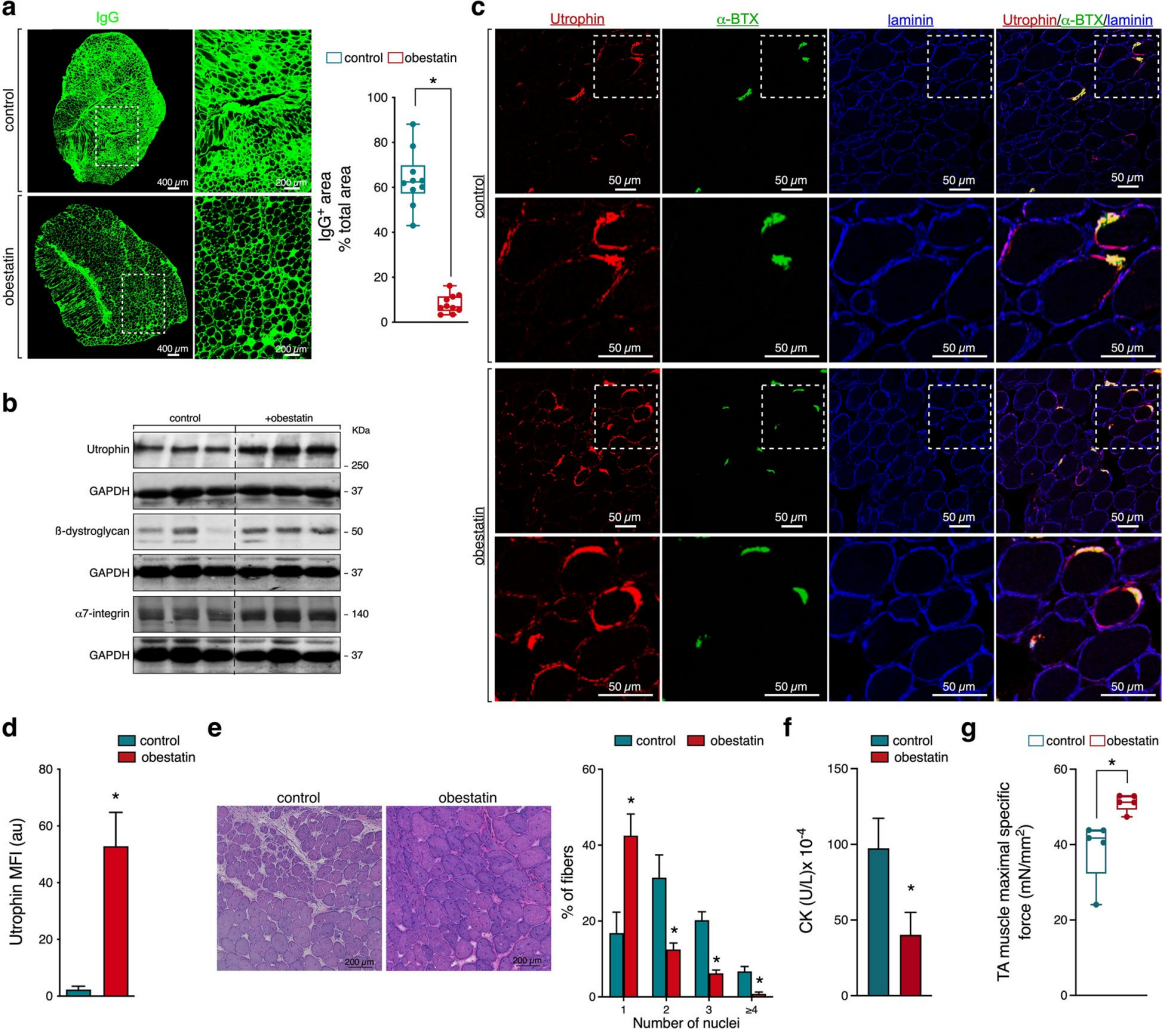
Obestatin ameliorates muscle damage in *mdx* mice. **a** Representative images of IgG staining and laminin of control and obestatin-treated TA muscles at 30 days. Line is set at median (*P < 0.05). **b** Expression analysis of utrophin, ß-dystroglycan, and α7-integrin, in TAs after intramuscular injection of obestatin or control at 30 days. **c** Representative immunofluorescence images of obestatin-treated or control TA muscles at 30 days showing utrophin, neuromuscular junctions (α‐BTX), and laminin expression. **d** The changes in MFI of ubiquitin are shown. Data were expressed as mean ± SEM (*P < 0.05). **e** Representative haematoxylin and eosin (HE) images of transversal cross-section of obestatin-treated TA muscles or control in TA muscles from *mdx* mice at 30 days. *Right panel*, quantification of fibres with centralized nuclei in obestatin- or control-treated TAs. Data are shown as mean ± SEM (*P < 0.05). **f** Serum CK levels after 30 days of treatment with vehicle or obestatin. Data are represented as mean ± SEM (*P < 0.05). **g** Effect of intramuscular injection of obestatin or vehicle on maximal specific force evaluated in TAs at 30 days. Line is set at median (*P < 0.05)

Because the reestablishment of mitochondrial homeostasis and autophagy converge on skeletal muscle remodelling [16], we assessed the contribution of these different routes to the extent of damaged myofibres in *mdx* mice. Consistent with our previous work, obestatin treatment decreased number of IgG-positive fibres supporting a reduction in muscle damage (Fig. 4a). Up‐regulation of utrophin, ß-dystroglycan and α7-integrin by obestatin led to stabilization of the sarcolemma (Fig. 4b). In fact, immunofluorescence analysis of obestatin-treated TAs showed elevated expression of utrophin (Fig. 4c,d). Furthermore, co-staining with a laminin antibody (sarcolemma), or α-bungarotoxin (α-BTX; neuromuscular junction) exhibited utrophin localization in the extrasynaptic compartment of the sarcolemma (Fig. 4c). The percentage of regenerated fibres with centralized nuclei in the TA muscle of obestatin-treated *mdx* mice was reduced (Fig. 4e). Furthermore, obestatin showed to reduce the creatine kinase (CK) in *mdx* animals (Fig. 4f). These findings reflected the protective action of obestatin against muscle damage. The overall effect is a functional benefit on the treated muscles, evidenced by the significant increase in the mean tetanic specific force in obestatin-treated muscles as compared to control TAs (Fig. 4g).

### PPP3 as signalling node to trigger the activation of slow-oxidative muscle development in response to obestatin

**Fig. 5:**
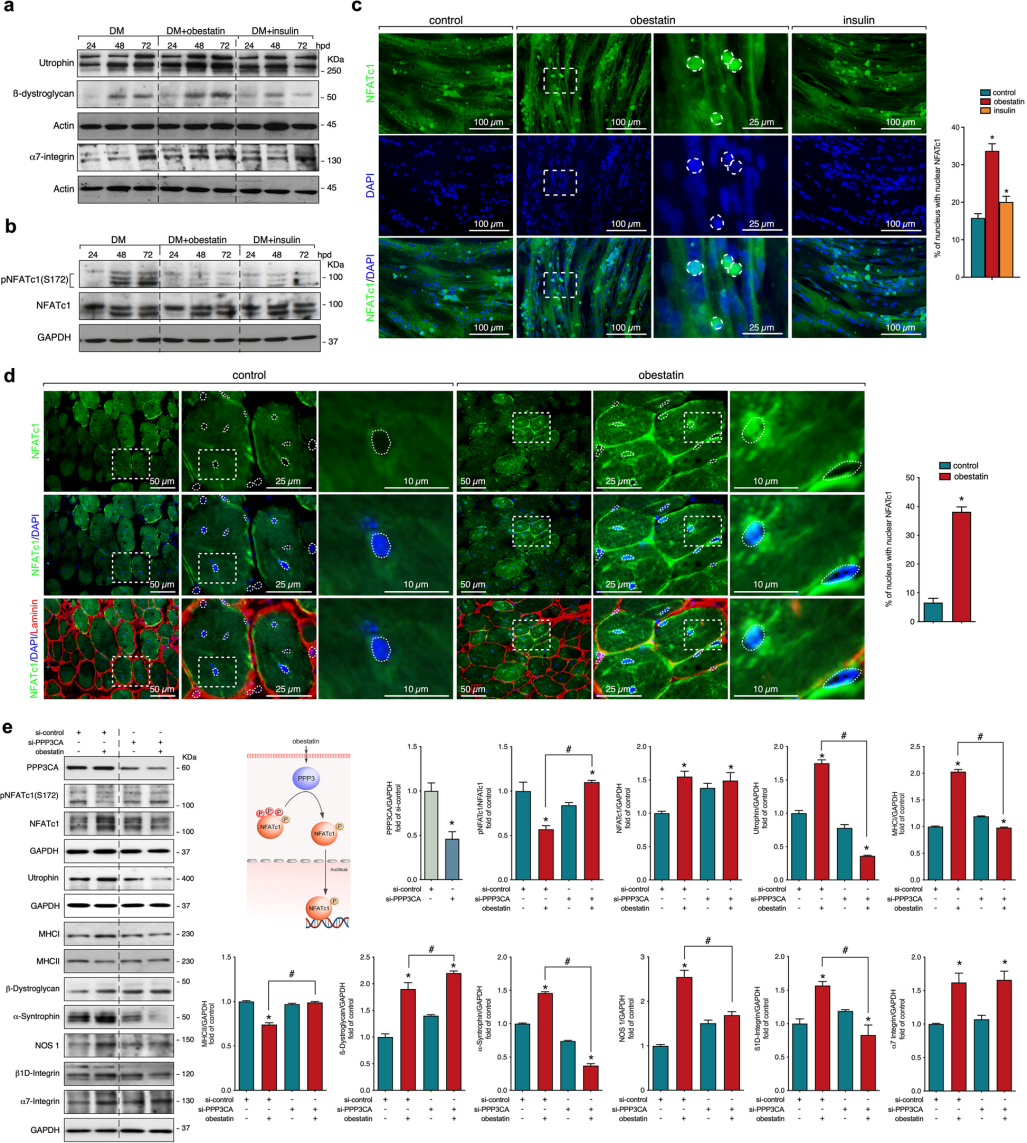
Obestatin promotes activation of NFTAc1. **a** Comparation of the effect of obestatin (10 nM) and insulin (1.72 µM) on utrophin, ß-dystroglycan, and α7-integrin protein expression on differentiating human DMD cells. **b** Analysis of pNFATc1(S172) and NFATc1 after obestatin (10 nM) or insulin (1.72 µM) administration on differentiating human DMD cells. **c** Representative immunofluorescence images of obestatin- or control-treated human DMD myotubes showing NFATc1 cellular location. *Right panel*, measurement of the number of nuclei showing NFATc1 location. Data are shown as mean ± SEM (*P < 0.05). **d** Representative images of vehicle- and obestatin-treated TAs showing NFATc1 expression. *Right panel*, measurement of the number of nuclei showing NFATc1 location. Data are shown as mean ± SEM (*P < 0.05). **e** Immunoblot analysis of PPP3 catalytic subunit (PPP3CA), pNFATc1(S172), NFATc1, utrophin, slow-MHC, fast-MHC, β-dystroglycan, α-syntrophin, NOS1, β1D-integrin, and α7-integrin in extracts of DMD cells transfected with control or PPP3CA siRNAs after obestatin treatment (10 nM). The inset shows Proposed model by which obestatin signalling triggers PPP3 to regulate the transcriptional activity of NFATc1. Data were expressed as mean ± SEM (*n* = 3 per group; *,^#^ *P* < 0.05)

**Fig. 6:**
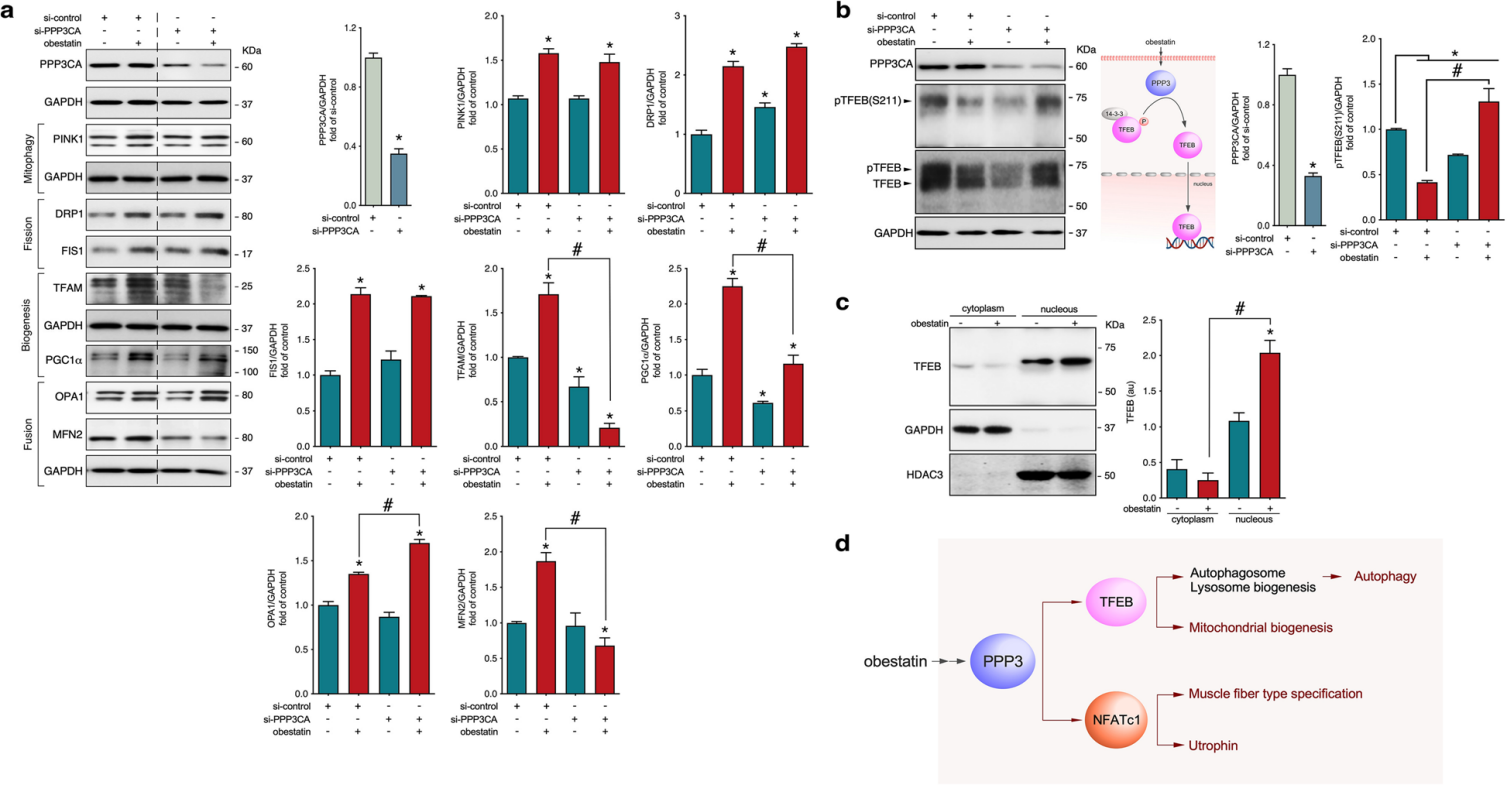
Obestatin promotes activation of mitochondrial homeostasis via calcineurin and TFEB activity. **a** Immunoblot analysis of PPP3CA, PINK1, DRP1, FIS1, TFAM, PGC1a, OPA1, and MFN2 in extracts of DMD cells transfected with control or PPP3CA siRNAs after obestatin treatment (10 nM). **b** Immunoblot analysis of PPP3CA, pTFEB(S211), and TFEB in extracts of DMD cells transfected with control or PPP3CA siRNAs after obestatin treatment (10 nM). The inset shows the proposed model by which obestatin signalling triggers PPP3 to regulate the transcriptional activity of TFEB. **c** Analysis of nuclear translocation of TFEB after obestatin treatment (10 nM) in human DMD myotubes. In A-C, data were expressed as mean ± SEM (*n* = 3 per group; *,^#^ *P* < 0.05). **d** Proposed model by which obestatin signalling triggers PPP3, NFATc1 and TFEB activity to regulate mitochondrial homeostasis, autophagy, muscle fibber type specification and sarcolemma repair obestatin signalling under dystrophic conditions

We confirmed the action of obestatin on the expression of sarcolemma adhesion protein components in human DMD myotubes. Immunoblot analyses revealed that obestatin elevated utrophin, ß-dystroglycan, and α7-integrin protein expression as compared with untreated human DMD cells (Fig. 5a). In this model, we attempted to establish a connection between the utrophin expression and the obestatin signalling network. In particular, we explored the activation of nuclear factor of activated T-cells 1 (NFATc1) signalling pathway in the specification of the slow myofibre phenotype and utrophin expression [20]. In contrast to untreated cells, obestatin showed a striking decrease in NFATc1 phosphorylation at S172 [pNFATc1(S172)], an effect also observed in insulin-treated cells (Fig. 5b). Immunofluorescence was used to visualise the location of NFATc1 protein in human DMD myotubes. In untreated cells, NFATc1 was mainly present in the cytoplasm (Fig. 5c). By contrast, obestatin increased the NFATc1 localisation in the nucleus (Fig. 5c). This action was also observed in insulin-treated cells, although to a much lesser extent (Fig. 5c). In this regard, obestatin promoted nuclear translocation of NFATc1 in TA from *mdx* mice (Fig. 5d). NFATc1 is one of main target for protein phosphatase 3 (PPP3, also known as calcineurin), a Ca^2+/^calmodulin-dependent serine/threonine phosphatase that is known for its role in promoting the oxidative metabolism in skeletal muscle [21]. PPP3 function was investigated using short interfering RNA (siRNA) targeting PPP3 catalytic subunit alpha (PPP3CA; si-PPP3CA) in human DMD cells. Knockdown of PPP3CA reduced protein levels of utrophin, α-syntrophin, nitric oxide synthase 1 (NOS1), and β1D-integrin (Fig. 5e). By contrast, the levels of β-dystroglycan and α7-integrin were unaffected (Fig. 5e). Analysis of MHCI (slow-MHC) showed that obestatin-activated increase in expression was cancelled when PPP3CA was silenced, contrary to that observed with MHCII (fast-MHC; Fig. 5e). Knockdown of PPP3CA impaired NFATc1 phosphorylation at S172 [pNFATc1(S172)] by obestatin with no effect on NFATc1 protein level (Fig. 5e). PPP3 may also act as regulator of mitochondrial dynamic [21]. In fact, PPP3CA-depleted cells exhibited a significant decrease in the levels of TFAM, PGC1α and MFN2 after obestatin treatment but did not alter PINK1, DRP1, FIS1 or optic atrophy 1 (OPA1) expression (Fig. 6a). Given the role of transcription factor EB (TFEB) as PPP3 substrate in the regulation of autophagy, lysosomal and mitochondrial biogenesis [22, 23], we investigated the regulation of TFEB protein in PPP3-depleted cells treated with obestatin. TFEB showed a reduction in the mobility of the band-shift after obestatin treatment, in agreement with decreased TFEB phosphorylation contrary to what occurred in PPP3CA knockout cells (Fig. 6b). Indeed, obestatin decreased the phosphorylation of TFEB at S211 which was reversed in cells transfecting with siPPP3 (Fig. 6b). We further evaluated the localization of TFEB through nucleus-cytoplasmic fractionation in human DMD cells (Fig. 6c). Obestatin treatment translocated TFEB to the nuclear fraction, with almost none remaining in the cytoplasmic compartment (Fig. 6c). Therefore, obestatin activates PPP3, which dephosphorylates NFATc1 and TFEB, leading to nuclear localization where they promote the expression of genes related to the slow-oxidative phenotype (Fig. 6d).

## Discussion

In this study, we combine both experimental animal and human cell-based models of the disease to show that obestatin positively impacts muscle mitochondrial health cycle, ameliorates DMD phenotype, and improves skeletal muscle physiological function. Treatment with obestatin up-regulates the master regulator of mitochondrial biogenesis, PGC-1⍺, to promote the expression of nuclear-encoded mitochondrial proteins, including TFAM. Obestatin is a nexus molecule to regulate mitochondrial degradation due to its regulation of autophagy and mitophagy markers. Obestatin promotes autophagy flux via AMPK to phosphorylate unc-51 like autophagy activating kinase 1(ULK1) at S555 to trigger the phosphorylation of Beclin-1 at S15, a critical step required for the autophagy initiation and endocytic trafficking. Mitophagy can occur by means of PINK1, which regulates PARKIN activity, among other proteins, to trigger mitochondrial removal. Obestatin coordinates the expression of OPA1 and MFN2 key molecular responsible for initiating mitochondrial fusion. In addition, obestatin activation contributes to mitochondrial fragmentation by upregulation of DRP1 and FIS1. Homeostatic coordination of mitochondrial dynamics and turnover controls the establishment of oxidative muscle fibres. In fact, long-term administration of obestatin promoted mitochondrial biogenesis, as evidenced by an increase in the mitochondrial content delineating the abundance of oxidative (expressing MHCI) muscle fibres. Remarkably, obestatin contributes to the maintenance of proper muscle morphology through the up-regulation of utrophin, β-dystroglycan, α-syntrophin, and α7β1-integrin proteins thereby facilitating sarcolemma stability and resistance and reducing muscle damage. These observations correlate with the reduction in the serum CK levels in treated *mdx* animals. The positive impact on mitochondrial homeostasis along with muscle remodelling capacity translates into improved muscle function in terms of specific force production. This finding represents a substantial advance in our understanding of the complex process of skeletal muscle remodelling under dystrophic conditions. It also identifies a potential candidate for combined therapeutic approaches, allowing an improvement of muscle stability and function that will increase the efficiency of gene and cell therapies.

In our opinion, the most straightforward explanation for the muscle protective effect of obestatin would be the restoration of muscle homeostasis and activation of membrane repair via of the calcium-dependent phosphatase PPP3 [24]. PPP3 dephosphorylates TFEB serine residues, which are crucial in determining TFEB subcellular localization, hence facilitating its nuclear translocation where it induces the transcription of target genes [22, 25–27]. In particular, TFEB is a principal regulator of set of genes related to glucose homeostasis, lysosome and mitochondrial biogenesis, which are essential for supplying the energy required to sustain muscular contraction [28]. Proper reactivation of mitochondrial homeostasis in dystrophic muscles, e.g. mitophagy, would eliminate dysfunctional mitochondria, restore cells with functional organelles, and ameliorate the symptoms of DMD. In parallel, PPP3 promotes the nuclear translocation of NFATc1, master regulator of muscle-related slow oxidation genes (MHCI, myoglobin, troponin C1, and PGC1α) [29, 30]. Indeed, our results reveal that in obestatin-treated muscles, the number of MHCI myofibres increased concurrently with the decrease of the MHCII myofibres (MHCIIb, and MHCIIx), whereas the average number of myofibres remains unaltered. Additionally, obestatin upregulates utrophin expression, achieving proper membrane localization. This result is in accordance with the activation of the oxidative myogenic programme via NFATc1 and PGC-1α signalling [31–33]. Importantly, these pathways are implicated in the control of components of utrophin glycoprotein complex (UGC), i.e. α-syntrophin, ß-dystroglycan and NOS1, and α7β1 integrins, elements aligned aligning with improved restoration of the UGC [34, 35]. Thus, transcription factors of the NFATc1 and TFEB families serve as endpoints for the obestatin-related signalling pathways whereby PPP3 controls muscle architecture and homeostasis.

Administration of obestatin not only alleviate DMD disease symptoms but also the muscle strength of *mdx* mice. This evidence reinforces the potential for translation into humans. Gene- and cell-based therapies represent a promising hope for targeting the genetic defects of DMD by repairing the mutated dystrophin gene or mRNA [36]. Despite the potential held by these therapy options, they can only repair one or a subset of mutations at a time and, hence, remain ineffective [37, 38]. In addition, it is uncertain whether correction of the primary disease-causing mutation corrects the associated secondary pathogenic symptoms [1, 39]. Indeed, recent studies have proven the synergistic effect of combined obestatin and cell-based treatment in mice [40]. Finally, gene therapy can use either direct delivery of the product, which implies repeated treatments, or viral vectors such as AAVs, which ensure long-term expression. Viral genomes, and thus transgenes or correcting oligonucleotides, are often lost when they hit a fibre that will degenerate soon after infection, thus decreasing the efficiency of these therapeutic strategies [41]. It is thus essential to develop combined therapies aiming at improving muscle integrity and function before applying any gene therapy using viral. This has been demonstrated combining both direct delivery and viral delivery [42]. The importance of combinatorial therapies is highlighted by our work, which identifies a clinically relevant pharmacological approach aims at optimising the treatment efficacy for muscle diseases [9]. While obestatin has been shown to be effective as a monotherapy, its applicability as a combined therapy offers the possibility of improving muscle quality and slowing disease progression as much as possible, in parallel with dystrophin restoration.

At this point, it is important to mention that the *mdx* mouse is the model of choice in DMD research due the simplicity and cost-effectiveness. However, this convenience is countered by the fact that *mdx* mouse mimics some symptoms but is less severe than human pathology. Furthermore, the role of obestatin was evaluated in a human in vitro model, but it is questionable whether this phenotype is representative of phenotypes of all types of mutations in DMD patients. Therefore, it would be important to examine the effect of obestatin in other animal and in vitro systems. Despite these aspects, we believe that our work constitutes a significant advance towards understanding the role of obestatin in dystrophic muscle, as well as the therapeutic potential that obestatin may have in DMD-associated myopathy.

This study uncovers a novel mechanism associated to obestatin signalling where the coordinated activation of TFEB and NFATc1 by PPP3 participates in restoration of muscle homeostasis and membrane repair under dystrophic conditions. Therefore, obestatin offers unique opportunities for muscle regenerative medicine, either as a single therapy or in combination with others.

## Materials and methods

### Materials

Human and mouse obestatin were purchased from BCN Peptides (Barcelona, ES). The antibodies used are specified in Table 1. All additional chemical reagents were purchased from Sigma Chemical Co (St. Louis, MO, US) unless stated otherwise.

**Table 1:** Primary antibodies. Relation of the primary antibodies used in the different analyses performed in this work

Primary antibody	Supplier	Reference	Use	Dilution
Actin	Abcam	ab1801	WB	1:1000
α-Bungarotoxin (α-BTX), Alexa Fluor™ 488 conjugate	Invitrogen	B13422	IF	1:500
AMPKα	Cell Signaling	2603	WB	1:1000
PPP3CA	Invitrogen	PA5-82,471	WB	0,2 µg/ml
DRP1	Cell Signaling	8570	WB	1:1000
β-dystroglycan	Santa Cruz	sc-33702	WB, IF	1:1000
FIS1	Cell Signaling	32,525	WB	1:1000
GAPDH	Invitrogen	PA1-987	WB	1:5000
Goat Anti-Mouse IgG H&L (FITC)	Abcam	ab6785	IF	1:200
HDAC3	Cell Signaling	3949	WB	1:1000
α7-integrin	Abcam	ab203254	WB, IF	1:500
β1D-integrin	Abcam	ab8991	WB	1:400
Laminin	Dako	Z0097	IF	1:200
LAMP2	Santa Cruz	sc-18822	IF	1:500
LC3	Cell Signaling	12,741	WB	1:1000
LC3B	Cell Signaling	2775	IF	1:1000
MFN2	Cell Signaling	9482	WB	1:1000
MHCI	DSHB	BA-F8	IF	2 µg/ml
MHCIIa	DSHB	SC-71	IF	2 µg/ml
MHCIIb	DSHB	BF-F3	IF	2 µg/ml
MHCIx	DSHB	6H1	IF	2 µg/ml
NFATc1	R&D	AF5640	WB, IF	1:500
NOS1	Santa Cruz	sc-5302	WB	1:500
OPA1	Cell Signaling	80,471	WB	1:1000
PARKIN	Cell Signaling	2132	WB	1:750
PGC1α	Invitrogen	PA5-72,948	WB	1:1000
pAMPKα (T172)	Cell Signaling	2535	WB	1:1000
pNFACTc1(S172)	R&D	MAB5640	WB	1:500
pTFEB(S211)	Cell Signaling	37,681	WB	1:1000
PINK1	Santa Cruz	sc-517353	WB	1:500
p62	Cell Signaling	5114	WB, IF	1:2000
α-syntrophin	Santa Cruz	sc-166634	WB	1:300
TFAM	Santa Cruz	sc-166965	WB	1:500
TFEB	Cell Signaling	37,785	WB	1:1000
TOM20	Santa Cruz	sc-17764	WB	1:1000
Ubiquitin	Santa Cruz	sc-166553	IF	1:200
Utrophin	Novocastra	NCL-DRP2	IF	1:200
VDAC	Santa Cruz	sc-390996	WB	1:500
Secondary antibody	Supplier	Reference	Use	
Peroxidase AffiniPure Goat Anti-Rabbit IgG (H + L)	Jackson ImmunoResearch	111–035-003=	WB	1:30,000
Peroxidase AffiniPure Goat Anti-Mouse IgG (H + L)	Jackson ImmunoResearch	115–035-003	WB	1:20,000
Peroxidase AffiniPure Donkey Anti-Goat IgG (H + L)	Jackson ImmunoResearch	705–035-003	WB	1:20,000
Goat anti-Rabbit IgG (H + L) Highly Cross-Adsorbed Secondary Antibody, Alexa Fluor™ 488	Invitrogen	A-11034	IF	1:500–1:1000
Goat anti-Mouse IgG1 Cross-Adsorbed Secondary Antibody, Alexa Fluor™ 594	Invitrogen	A-21125	IF	1:500–1:1000
Goat anti-Mouse IgG (H + L) Highly Cross-Adsorbed Secondary Antibody, Alexa Fluor™ 488	Invitrogen	A-11029	IF	1:500–1:1000
Goat anti-Mouse IgG2b Cross-Adsorbed Secondary Antibody, Alexa Fluor™ 594	Invitrogen	A-21145	IF	1:500–1:1000
Goat anti-Mouse IgM (Heavy chain) Cross-Adsorbed Secondary Antibody, Alexa Fluor™ 594	Invitrogen	A-21044	IF	1:500–1:1000
Goat anti-Rabbit IgG (H + L) Cross-Adsorbed ReadyProbes™ Secondary Antibody, Alexa Fluor™ 594	Invitrogen	R37117	IF	1:500–1:1000
Donkey anti-goat IgG Alexa 488	Abcam	ab150129	IF	1:500–1:1000

*WB *Western blot, *IF* Immunofluorescence

### Mouse studies

Male C57BL/10 J (C57 mice) and C57BL/10ScSn-Dmdmdx/J (DMD model mice; *mdx*) mice were purchased at The Jackson Laboratory (Bar Harbor, ME, USA). For protein expression assays, 8-, and 18-week-old male C57 (*n* = 5 per group) and *mdx* (*n* = 5 per group) mice were used. To evaluate the effects of exogenous obestatin in dystrophic muscles, 8-week-old male *mdx* mice were utilized (*n* = 5 per group). Obestatin (500 nmol/kg body weight), a dose previously used [13], or vehicle control [PBS (pH 6.3)] was administrated via injection into the tibialis anterior (TA) every 72 h during 30 days (*n *= 5 per group) [16, 40]. After 30 days, mice were anaesthetised by intraperitoneal injection of xylazine (10 mg/kg) and ketamine (100 mg/kg) [16]. Skeletal muscles were dissected from euthanized mice and flash frozen in nitrogen-cooled isopentane for subsequent analysis. The protocols for animal experiments were approved by the University of Santiago de Compostela Animal Care Committee according to the guidelines of the Spanish Royal Decree 53–2013, Directive 2010–63-EU, and FELASA Guidelines (Approval nº. 15,010/17/005).

### Measurements of muscle force in vivo

Muscle force was evaluated in living animals as previously described [13]. The contractile performance of the TA was measured in anaesthetised mice using the 1305 A Whole Animal System (Aurora Scientific, Inc., ON, Canada). Electrical stimulation of the common peroneal nerve by an electrode placed in the popliteal fossa was used to evoke TA contractions. Force-frequency curves were obtained by stepwise increasing the stimulation frequency. Pauses were included between stimuli to prevent fatigue-related effects.

### Blood measurements

Peripheral blood was extracted via inferior vena cava puncture under general anaesthesia at the end of the treatment. CK analysis was performed by the Laboratorio Central at the Hospital Clínico Universitario de Santiago de Compostela (Santiago de Compostela, ES).

### Cell culture studies

Human DMD14 myogenic clonal cell line (DMD cells) was acquired from the Centre for Research in Myology (Paris, FR). The platform of immortalization developed the isolation and immortalization from a biopsy obtained through MYOBANK (EU network EuroBioBank), in accordance with European recommendations and French legislation. DMD myoblasts were isolated from paravertebral muscle biopsy obtained from 14-year-old male DMD patient (duplication of exon 2) [16, 43, 44]. Myoblasts were cultivated in growth medium (GM) containing Medium 199: DMEM (1:4, v:v; Lonza, Pontevedra, ES) supplemented with 20% FBS (v/v), 25 µg/µL fetuin, 5 ng/mL hEGF, 0.5 ng/mL bFGF, 0.2 µg/mL dexamethasone and 50 µg/mL gentamycin (Invitrogen, ThermoFisher Scientific; MA, US). Myotube differentiation was initiated at 90% confluence by switching to differentiation medium [DM; DMEM supplemented 50 µg/mL gentamycin (Invitrogen)] during 3 days.

### Muscle staining

Muscle samples were embedded in tragacanth gum and snap frozen in nitrogen-cooled isopentane. Serial Sections (8 µm thick) were stained with haematoxylin and eosin (HE) or SDH according to standard protocols. For immunofluorescence analysis, skeletal muscle sections were permeabilized and blocked with PBST [1% Tween-20 (v/v), 1% Triton X-100 (v/v), 5% heat inactivated goat serum (v/v), 0.2% BSA (w/v) in PBS] for 30 min (min). Subsequently, sections were incubated with primary antibodies diluted in PBST overnight at 4 °C, followed by a washing step with PBS and a second incubation with secondary antibody for 45 min at 37 °C. For analysis of cultured cells, the DMD myoblasts were cultured and differentiated into myotubes on coverslips. The myotubes were then fixed with ethanol, washed, permeabilised and blocked with PBST. Samples were subsequently incubated with diluted primary antibody in PBST overnight at 4 °C. The cells were then washed and incubated with secondary antibody for 1 h at room temperature. Table S1 shows the primary and secondary antibodies used in this work (supplementary information). Cell nuclei were counterstained with 4′,6-diamidino-2-phenylindole (DAPI) (Invitrogen). Images were collected blind (> 3 images per condition) from three independent repeats. The digital images were acquired with a Leica TCS-SP8 confocal microscope (Leica Microsystems, Heidelberg, DE).

### Western blot analyses

Protein was isolated from cells or tissue using ice-cold RIPA buffer [50 mmol/l Tris–HCl (pH 7.2), 1 mmol/l EDTA, 150 mmol/l NaCl, 1% NP-40 (v/v), 0.25% Na-deoxycholate (w/v), phosphatase and protease inhibitor cocktails]. For nuclear-cytoplasmic cell fractionation, the cells were lysed in PBS containing 0.1% NP-40 (w/v) and phosphatase and protease inhibitor cocktails. The lysates were subjected to centrifugation at 10.000 rpm for 10 s. The supernatant (cytosolic fraction) was separated from the pellet (nuclear fraction). The pellet was subsequently washed once and lysed in SDS sample buffer. QuantiProTM BCA assay kit was used to quantify the protein concentration and the lysates were resolved by SDS-PAGE. Primary and secondary antibodies are detailed in Table S1 (supplementary information). Immunoreactive bands were detected by enhanced chemiluminescence (Pierce ECL Western Blotting Substrate; Thermo Fisher Scientific, Rockford, IL, US). Immunoblots were quantified by densitometry using NIH Image software, ImageJ 1.5b (National Institutes of Health, Bethesda, MD, US).

### Mitochondrial analysis

Human DMD myoblast cells were seeded and differentiated on coverslips, and live myotubes were loaded with MitoTracker Deep Red® (Invitrogen) after obestatin (10 nM) or vehicle treatment (24 h) following the instructions provided by the manufacturer.

### Small interfering RNA-mediated gene silencing

To knockdown PPP3 expression in human DMD cells, siRNA specifically targeting human PPP3 catalytic subunit alpha (sc-36303, Santa Cruz Biotechnology, CA, US) was used. An ON-TARGETplus nontargeting siRNA (Dharmacon) was utilized as control. Cells were transfected with Lipofectamine 2000 (Invitrogen), following the instructions provided by the manufacturer. Cell viability was measured after transfection using a trypan blue staining protocol (70–80% after transfection). Silencing efficiencies obtained herein ranged 55–67%.

### Statistical analyses

All data were represented as mean values ± SEM. Statistical differences among the groups were evaluated by GraphPad Prism (version 10; GraphPad Software, San Diego, CA, USA). Both parametric and non-parametric analyses were applied, in which the Mann–Whitney rank sum test (Mann–Whitney U-test) was chosen for samples on a non-normal distribution, while a two-tailed t-test was used for samples with a normal distribution, respectively. *P* < 0.05 was considered as statistically significant.

## Data Availability

All data are available from the corresponding authors upon request.
